# Human Tick-Borne Encephalitis, the Netherlands

**DOI:** 10.3201/eid2301.161405

**Published:** 2017-01

**Authors:** Vishal Hira, Barry Rockx

**Affiliations:** St. Antonius Hospital, Nieuwegein, the Netherlands (V. Hira);; National Institute for Public Health and the Environment, Bilthoven, the Netherlands (B. Rockx)

**Keywords:** tick-borne encephalitis, tick-borne, tick, encephalitis, the Netherlands, Sweden, incubation period, antibodies, laboratory diagnosis, meningitis/encephalitis, vector-borne infections, intrathecally produced antibodies, IgG, IgM, tick bite, flaviviruses

**To the Editor:** We read with interest the report by Henningsson et al. (*1*) on a recent human case of tick-borne encephalitis (TBE) in Sweden. This case has multiple similarities to a case in the Netherlands. Until recently, TBE virus (TBEV) was not thought to be present in the Netherlands. However, detection of TBEV in ticks from a forested area in the Netherlands (S. Jahfari et al., Centre for Infectious Disease Control (CIb), RIVM, the Netherlands, pers. comm., 2016, Jun 30) led to identification of an autochthonous case of TBE (*2*). Of note, the presumed incubation period in both cases was very short, only 2 days. In both cases, quantitative reverse transcription PCR of a tick removed from the patient’s leg showed high levels of TBEV RNA. Another similarity between the cases was the absence of intrathecally produced TBEV antibodies (no IgM and a negative antibody index for IgG for the case in the Netherlands) at hospital admission. Although 16% of TBE patients have no intrathecally produced TBEV antibodies at hospital admission, these antibodies are usually important for diagnosis (*3*). These cases underscore the point that absence of these antibodies does not rule out TBE. 

By the time patients undergo TBE testing, the virus is typically no longer detectable in clinical samples (*4*). Therefore, laboratory diagnosis is done on the basis of the presence of antibodies in blood. Flavivirus serologic testing is, however, challenging due to cross-reactivity between the flaviviruses. Thus, confirmation by virus neutralization test is typically required. 

These 2 studies (*1**,**2*) show that virus detection in ticks from TBE patients can be useful for the definitive diagnosis of TBE. However, in low-prevalence settings, it would be inefficient to test ticks from all patients with tick bites. In these cases, ticks should be tested only when TBE is suspected on the basis of compatible clinical symptoms and laboratory findings, such as positive TBEV serologic test results.
